# In memoriam: Paul Fisch (1959–2018) our dear friend, pioneer of γδ T cell research, esteemed scientist, and dedicated clinician

**DOI:** 10.1002/cti2.1048

**Published:** 2019-04-24

**Authors:** Gabrielle M Siegers, Miroslav Malkovsky

**Affiliations:** ^1^ Department of Oncology University of Alberta Edmonton AB Canada; ^2^ School of Medicine and Public Health University of Wisconsin Madison Madison WI USA

## Abstract

In the wake of the sudden passing of Professor Paul Fisch, colleagues, collaborators and friends shared their thoughts on Paul's significant contributions to γδ T cell research and the scientific community at large.

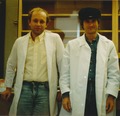

In 1968, shortly after the Russian invasion and occupation of Czechoslovakia, Dr Arthur Fisch, an Auschwitz survivor, relocated with his wife and 8‐year‐old Paul from Bratislava to Bad Neustadt an der Saale (West Germany). Paul studied medicine with an internship in Neurology in Cologne. After residency in Internal Medicine in Würzburg, Paul joined Paul Sondel's group at the Carbone Cancer Center at the University of Wisconsin in Madison, as a Leukemia Society of America Fellow. He was then awarded a German Research Foundation scholarship to work with Terry Rabbits at the Laboratory of Molecular Biology in Cambridge, UK. Paul returned to Germany as a group leader and Leukemia Society Fellow in the Department of Hematology and Oncology at the University of Freiburg, completing a Habilitation degree in Immunology under Thomas Boehm. Paul was then awarded the prestigious Heisenberg scholarship, joining Hans‐Georg Rammensee's department at the University of Tübingen, where he completed a Habilitation in Immunology and Molecular Genetics. There he also received the Artur‐Pappenheim‐Award from the German Society of Hematology and Medical Oncology. In 1998, Paul returned to Freiburg as Professor of Molecular Pathology at the Institute for Clinical Pathology. In 2016, on his return from a scientific trip to Buenos Aires, Paul suffered a fall causing two vertebral fractures. Subsequent medical intervention and further complications thereof ultimately led to his death on 26 November 2018.

Paul was a true pioneer of γδ T cell research, interested in multiple facets of the cells’ biology. Drawing scientific insight from meticulous investigation of human clinical data and cell culture systems, Paul made profound contributions to our understanding of γδ T cells, and to cancer immunology more broadly.

After the wholly unanticipated discovery of the TCRγ chain^1^ and the subsequent discovery of γδ T cells,^2^, ^3^ some immunologists doubted that γδ T cell antigen recognition would differ from that of αβ T cells. However, word emerged from the Sondel lab in Wisconsin that – incontrovertibly – peripheral blood γδ T cells kill haematological malignancies in the absence of known MHC I or MHC II elements. The young clinician scientist Paul Fisch spearheaded these studies, bringing his findings to the 1990 International Workshop on the ‘Specificity and Function of γδ T cells’ at Schloss Elmau in Bavaria, Germany.

Paul's time in Madison was fundamental to both his professional and personal life. Not only Paul's scientific discoveries,^4^, ^5^, ^6^ but also his friendships from those years would carry on for the rest of his life (Figure 1). In his seminal paper on γδ T cell specificity, Paul was one of the first to describe the molecular mimicry that allows γδ T cells to be shaped by infections but also to recognize tumour cells.^5^ In addition, Paul showed that γδ T cells can be subdivided into distinct subsets according to their cytotoxic responses,^6^ and that while inhibitory receptors typically found on NK cells likewise control γδ T cell cytotoxicity against Daudi lymphoma targets,^7^ γδ T cells are functionally distinct from NK cells.^4^, ^6^ These important discoveries set the landscape for a wave of studies about the role of NK receptors on γδ T cells, and their innate immune responses in tumour immunity,^8^, ^9^ which remains a very active research field.^10^, ^11^ With these early studies, Paul moved a large and important stone into place on the pathway to recognizing γδ T cell biology as unique and clinically profound. Indeed, Paul's work has proven prescient, as reflected in the wealth of strategies under development to harvest this potential. Biotech company founders in the γδ T cell space agree that Paul's discoveries provided scientific underpinnings that have enabled progress towards clinical trials. Beyond his early work, Paul continued to make crucial contributions as a sharp, fair reviewer and critical discussion partner for these companies. Naturally, Paul's research also continued in this vein, with recent papers on enhancement of tumour cell killing *via* manipulation of the γδ T cell antigen receptor 12, 13 and analysis of γδ T cells infiltrating triple‐negative breast cancers.^14^

**Figure 1:**
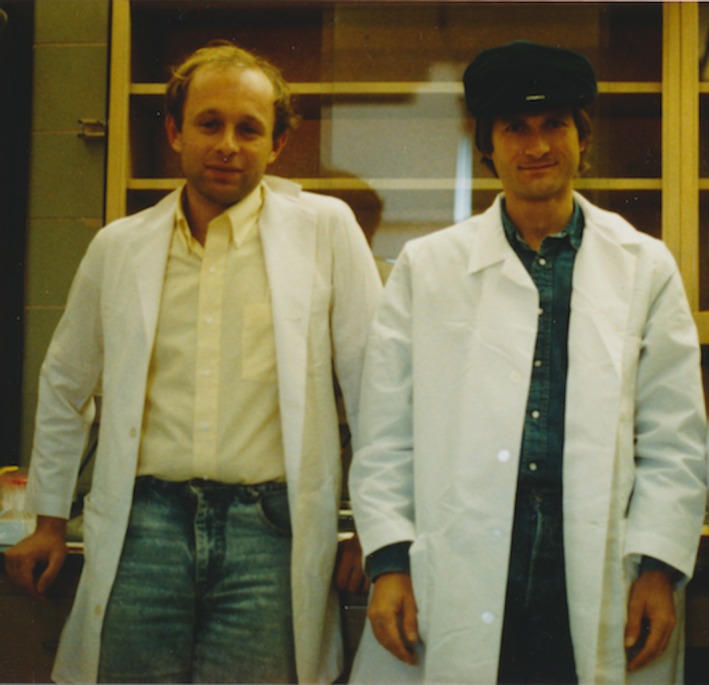
Paul Fisch and Mirek Malkovsky in Mirek's Lab in Madison, Wisconsin, in August 1989.

Paul not only thought about science, he also loved performing experiments! His keen interest in how natural phosphoantigens activate γδ T cells led Paul to bring a huge dry pellet of Daudi cells to Jean‐Jacques Fournié in Toulouse for chromatographic separation. Careful analyses revealed only trace amounts of these very labile metabolites, unfortunately an unpublishable result. Undeterred, the duo screened a collection of ~2000 natural product extracts that Jean‐Jacques had assembled during family holidays. Paul consistently observed activating compounds in these samples, pointing to ubiquitous stimuli for these unconventional T lymphocytes. More recently, while studying the diversity of γδ T cell expansions in immunodeficient patients and analysing the underlying mechanisms, Paul substantially improved the TCRγ and δ spectratyping method through intense laboratory experimentation.^15^

Paul was a central figure in the γδ T cell arena, highly respected for his honesty, work ethic and outstanding commitment to research. Paul's eminent role was honoured with his appointment as a chairman for the 2010 γδ T cell meeting in Kiel, Germany. Then, when the next organizers were unable to host the following conference, Paul stepped up and, together with Wolfgang Schamel, rescued the 2012 γδ T cell meeting on short notice. Paul wrote a conference grant application to the German Research Foundation that, hastily signed on the hood of Paul's car under a street lamp on a dark and drizzling Sunday evening, was thankfully successful!^16^

Paul was also important to the γδ T cell community on a personal level. Paul warmly welcomed researchers from other fields and befriended newcomers at γδ T cell conferences. Deeply committed to family, Paul would sometimes bring one of his children to γδ T cell meetings, and could carry on discussions about the unexpected characteristics and multifunctionality of γδ T cells while picking out colourful doughnuts!

Not without some measure of unforeseen disarray, Paul was a generous and reliable collaborator, and helped junior and senior researchers alike gain a step up into the γδ T cell world by freely sharing his expertise and support. Paul inspired a new generation of scientists to pursue greater knowledge of γδ T cells. Many scientists cloning γδ T cells use Paul's protocol,^4^ often accompanied by his personal guidance.

Paul also collaborated with clinicians outside of the γδ T cell field, studying patients with inherited immunodeficiencies and analysing the consequences of hypomorphic mutations in genes critical for VDJ recombination.^17^ This intriguing work was the beginning of several interesting discoveries in this area spanning over a decade, the most recent published in *Blood* in 2016.^18^ Paul's additional non‐γδ T cell studies included thymoma‐associated immunodeficiencies^19^ and hematopoietic cell transplantations.^20^

Paul was a quiet and humble person, with a demeanour that belied his vast intelligence. In his calm, friendly and reassuring way, he treated his conversation partners as equals, thereby instilling confidence in those who sought him out. It was easy to speculate and hypothesize with Paul, as he was an excellent listener, was very well read and his comments were thoughtful, pragmatic and to the point. Paul stood by his ideas, which collaborators and competitors alike sometimes found challenging, but they would always recognize the benefits of his input nonetheless. Paul's way of thinking might affectionately be described as a sort of ‘creative chaos’, as might his appearance, but his mind was sharp and analytical.

Paul was a loyal friend, and an encouraging and generous mentor. He was quirky, yet warm and open not only with friends, but also with collaborators and students; discussions often went beyond science, to include family, philosophy or politics, sometimes over döner kebab or cheesecake and fine coffee. Those who were close to him appreciated Paul's special sense of humour, humanity and kindness.

In his final year, Paul impressed all those around him with his energy and scientific drive, despite his failing health. Paul's unstoppable vision was to develop the use of γδ T cells for anticancer immunotherapy. As such, he mustered his remaining strength, will and passion to rally scientists to form a German consortium to analyse the role of γδ T cells in cancer, and submitted a funding proposal to the Deutsche Krebshilfe. When awarded an invitation to proceed, Paul fought his ill health to prepare for the defence *in viva voce*. With his iron will to advance science, Paul disregarded his deteriorating health, travelling to Bonn to defend his dream. On this unforgettable day, those with him shared moments of excitement, laughter, anger, eagerness and empathy. Despite his weakened state, Paul was following his passion and was therefore happy. He passed away a few days later, never having learned the outcome.

The news of Paul's death came as a shock to all who knew him. Paul was a rare combination of kindness of heart and keenness of mind, and we feel very lucky to have had him as a friend and colleague. He brought us an abundance of beauty, inspiration, originality, authenticity, kindness and love. We sincerely hope Paul's wife Anja, daughter Nora and sons David and Aron will find some comfort and solace in knowing what an indelible impression Paul left on so many of us. He did not need a long life for us to measure. It was, rather, we who needed his life to be longer, and though he is gone, his unconditional love for science will continue to inspire us.

## References

[1] Hayday AC , Saito H , Gillies SD *et al* Structure, organization, and somatic rearrangement of T cell gamma genes. Cell 1985; 40: 259–269. 3917858 10.1016/0092-8674(85)90140-0

[2] Brenner MB , McLean J , Dialynas DP *et al* Identification of a putative second T‐cell receptor. Nature 1986; 322: 145–149. 27183647

[3] Bank I , DePinho RA , Brenner MB *et al* Chess L . A functional T3 molecule associated with a novel heterodimer on the surface of immature human thymocytes. Nature 1986; 322: 179–181. 3487737 10.1038/322179a0

[4] Fisch P , Malkovsky M , Braakman E *et al* Gamma/delta T cell clones and natural killer cell clones mediate distinct patterns of non‐major histocompatibility complex‐restricted cytolysis. J Exp Med 1990; 171: 1567–1579. 2185329 10.1084/jem.171.5.1567 PMC2187884

[5] Fisch P , Malkovsky M , Kovats S *et al* Recognition by human V gamma 9/V delta 2 T cells of a GroEL homolog on Daudi Burkitt's lymphoma cells. Science 1990; 250: 1269–1273. 1978758 10.1126/science.1978758

[6] Fisch P , Oettel K , Fudim N *et al* MHC‐unrestricted cytotoxic and proliferative responses of two distinct human gamma/delta T cell subsets to Daudi cells. J Immunol 1992; 148: 2315–2323. 1532810

[7] Fisch P , Meuer E , Pende D *et al* Control of B cell lymphoma recognition via natural killer inhibitory receptors implies a role for human Vγ9/Vδ2 T cells in tumor immunity. Eur J Immunol 1997; 27: 3368–3379. 9464825 10.1002/eji.1830271236

[8] Fisch P , Moris A , Rammensee HG *et al* Inhibitory MHC class I receptors on gammadelta T cells in tumour immunity and autoimmunity. Immunol Today 2000; 21: 187–191. 10740240 10.1016/s0167-5699(99)01576-5

[9] Rothenfusser S , Buchwald A , Kock S *et al* Missing HLA class I expression on Daudi cells unveils cytotoxic and proliferative responses of human gammadelta T lymphocytes. Cell Immunol 2002; 215: 32–44. 12142034 10.1016/s0008-8749(02)00001-1

[10] Siegers GM , Dhamko H , Wang XH *et al* Human Vdelta1 gammadelta T cells expanded from peripheral blood exhibit specific cytotoxicity against B‐cell chronic lymphocytic leukemia‐derived cells. Cytotherapy 2011; 13: 753–764. 21314241 10.3109/14653249.2011.553595

[11] Silva‐Santos B , Strid J . Working in “NK Mode”: natural killer group 2 member D and natural cytotoxicity receptors in stress‐surveillance by γδ T cells. Frontiers in Immunol 2018; 9: 851. 10.3389/fimmu.2018.00851 PMC5928212 29740448

[12] Juraske C , Wipa P , Morath A *et al* Anti‐CD3 fab fragments enhance tumor killing by human γδ T cells independent of Nck recruitment to the gammadelta t cell antigen receptor. Frontiers in Immunol 2018; 9: 1579. 10.3389/fimmu.2018.01579 PMC6046647 30038626

[13] Dopfer EP , Hartl FA , Oberg HH *et al* The CD3 conformational change in the γδ T cell receptor is not triggered by antigens but can be Enforced to enhance tumor killing. Cell Rep 2014; 7: 1704–1715. 24857663 10.1016/j.celrep.2014.04.049

[14] Hidalgo JV , Bronsert P , Orlowska‐Volk M *et al* Histological analysis of gammadelta T lymphocytes infiltrating human triple‐negative breast carcinomas. Frontiers in Immunol 2014; 5: 632. 10.3389/fimmu.2014.00632 PMC4261817 25540645

[15] Christopoulos P , Bukatz D , Kock S *et al* Improved analysis of TCRgammadelta variable region expression in humans. J Immunol Methods 2016; 434: 66–72. 27109705 10.1016/j.jim.2016.04.009

[16] Silva‐Santos B , Schamel WW , Fisch P *et al* gammadelta T‐cell conference 2012: close encounters for the fifth time. Eur J Immunol 2012; 42: 3101–3105. 23255005 10.1002/eji.201270101

[17] Ehl S , Schwarz K , Enders A *et al* A variant of SCID with specific immune responses and predominance of gamma delta T cells. J Clin Invest 2005; 115: 3140–3148. 16211094 10.1172/JCI25221 PMC1242191

[18] Janda A , Schwarz K , van der Burg M *et al* Disturbed B‐lymphocyte selection in autoimmune lymphoproliferative syndrome. Blood 2016; 127: 2193–2202. 26907631 10.1182/blood-2015-04-642488

[19] Christopoulos P , Dopfer EP , Malkovsky M *et al* A novel thymoma‐associated immunodeficiency with increased naive T cells and reduced CD247 expression. J Immunol 2015; 194: 3045–3053. 25732729 10.4049/jimmunol.1402805

[20] Rathmann S , Keck C , Kreutz C *et al* Partial break in tolerance of NKG2A^‐^/LIR‐1^‐^ single KIR^+^ NK cells early in the course of HLA‐matched, KIR‐mismatched hematopoietic cell transplantation. Bone Marrow Transplant 2017; 52: 1144–1155. 28481352 10.1038/bmt.2017.81

